# Heterotopic ossification in primary total hip arthroplasty using the posterolateral compared to the direct lateral approach

**DOI:** 10.1007/s00402-021-03783-6

**Published:** 2021-02-03

**Authors:** J. H. J. van Erp, J. R. A. Massier, S. Truijen, J. E. J. Bekkers, T. E. Snijders, A. de Gast

**Affiliations:** 1 Clinical Orthopedic Research Center, mN, Zeist, The Netherlands; 2 Department of Orthopedic Surgery, Diakonessenhuis, Utrecht, The Netherlands

**Keywords:** Total hip arthroplasty, Periarticular ossification, PAO, Direct lateral approach, Posterior approach

## Abstract

**Purpose:**

Total hip arthroplasty (THA) is a successful procedure. However, in time, heterotopic ossification (HO) can form due to, amongst others, soft tissue damage. This can lead to pain and impairment. This study compares the formations of HO between patients who underwent either THA with the posterolateral approach (PA) or with the direct lateral approach (DLA). Our hypothesis is that patients who underwent THA with a PA form less HO compared to THA patients who underwent DLA.

**Methods:**

In this prospective cohort study, 296 consecutive patients were included who underwent THA. A total of 127 patients underwent THA with the PA and 169 with the DLA. This was dependent on the surgeon’s preference and experience. More than 95% of patients had primary osteoarthritis as the primary diagnosis. Clinical outcomes were scored using the Numeric Rating Scale (NRS) and Harris Hip Score (HHS), radiological HO were scored using the Brooker classification. Follow-up was performed at 1 and 6 years postoperatively.

**Results:**

Two hundred and fifty-eight patients (87%) completed the 6-year follow-up. HO formation occurred more in patients who underwent DLA, compared to PA (43(30%) vs. 21(18%), *p* = 0.024) after 6 years. However, the presence of severe HO (Brooker 3–4) was equal between the DLA and PA (7 vs. 5*,* *p* = 0.551). After 6 years the HHS and NRS for patient satisfaction were statistically significant higher after the PA (95.2 and 8.9, respectively) compared to the DLA (91.6 and 8.5, respectively) (*p* < 0.001 and *p* = 0.003, respectively). The NRS for load pain was statistically significant lower in the PA group (0.5) compared to the DLA group (1.2) (*p* = 0.004). The NRS for rest pain was equal: 0.3 in the PA group and 0.5 in the DLA group.

**Conclusion:**

THA with the PA causes less HO formation than the DLA.

**Trial registration:**

Registrated as HipVit trial, NL 32832.100.10, R-10.17D/HIPVIT 1. Central Commission Human-Related research (CCMO) Registry.

## Introduction

Total hip arthroplasty (THA) is a successful treatment for patients with hip osteoarthritis, resulting in less pain, a greater range of motion (ROM) and a considerably higher quality of life [1, 2]. However, in a minority of patients, peri-articular heterotopic ossifications (HO) develop postoperatively. These bony formations in the soft tissues of the operated hip can lead to pain and impairment of ROM [3, 4]. Especially severe HO can lead to significant disability [5].

Soft tissue damage caused by the surgery provides an environment that causes osteoblasts to arrive from mesenchymal cells, resulting in HO [3, 4]. Therefore, it is thought that increased soft tissue damage during surgery result in more HO [6]. Known risk factors for the formation of HO include male sex, smoking, a chronic infection, high number of operations, hypertrophic osteoarthritis, previous development of HO, posttraumatic arthritis, ankylosing spondylitis, diffuse hyperostosis, Paget’s disease, paraplegia or traumatic brain injury [7–11].

However, there is no consensus about the influence of different surgical approaches in THA on the formation of HO. A more extensive approach may cause more tissue damage which increases formation of HO [6, 12–14]. These studies compare anterior, anterolateral and direct lateral approaches. No information is available about HO formation after a direct lateral approach (DLA) compared to a posterolateral approach (PA) yet.

The aim of this study is to compare the presence and severity of HO after 1 and 6 years after THA between the DLA and the PA for THA.

## Materials and methods

### Study design

This prospective cohort study was performed at the Diakonessenhuis Hospital, a medium size general hospital in Utrecht/Zeist, the Netherlands. Ethical local institutional review board approval was obtained. Informed consent was acquired from all participating patients. Between 2009 and 2015, a total of 296 consecutive patients were included. All included patients are derived from two existing prospective THA cohorts of our hospital [15, 16]. All patients scheduled for primary THA between 20 and 85 years old with either primary osteoarthritis or secondary osteoarthritis due to congenital hip dysplasia, rheumatoid arthritis, avascular necrosis of the femoral head or trauma and who were willing to participate in this study were eligible. Patients with an ASA score > III were excluded, because both cohorts were established to describe long-term follow-up after THA. Baseline characteristics are shown in Table 1. More than 95% of patients had primary osteoarthritis as the primary diagnosis. The other patients were included after a fracture, because of secondary osteoarthritis, hip dysplasia or avascular necrosis of the femoral head. None of the included patients had one of the following risk factors for developing HO: diffuse osteopathic skeletal hyperostosis, Paget’s disease or paraplegia. We did not collect data on hypertrophic osteoarthritis or previous development of HO or hypertrophic.

**Table 1:** Patients’ characteristics

	PA (*n* = 127)	DLA (*n* = 169)	*p* value
Age, years (SD)	64 ± 7	65 ± 7	0.197
Gender, female (%)	85 (67)	121 (72)	0.387
Body Mass Index (SD)	26 ± 4	27 ± 4	0.769
Non-steroidal anti-inflammatory drug use* (%)	80 (64)	110 (66)	0.419

*Information was missing for 68 patients

Patients were scheduled for clinical and radiological follow-up after 1 and 6 years. At each follow-up HHS, NRS score for rest and load pain and patient satisfaction were documented, and antero-posterior radiographs of the pelvis in supine position were made. Follow-up was performed by two investigators (JHJE and TES). The primary outcome of this study is the prevalence of HO after 6 years. Secondary outcomes are the presence of HO after 1 year and clinical outcomes at both moments.

### Procedure

Critical aspects of the surgical procedure were standardized. The procedures were performed by seven orthopedic surgeons, each with vast experience in uncemented THA. The surgical approach used was dependent on the surgeon’s preference and experience. Alumina ceramic femoral prosthetic heads (BIONIT2, Mathys Ltd, Bettlach, Switzerland) of 28, 32 or 36 mm were used. An uncemented hydroxyapatite coated stem (Twinsys, Mathys Ltd, Bettlach, Switzerland) was implanted in all cases. All patients received an uncemented vitamin E blended HXLPE cup (RM uncemented monoblock pressfit Vitamys^®^ cup, Mathys Ltd, Bettlach, Switzerland) or a UHMWPE cup (RM uncemented monoblock pressfit^®^, Mathys Ltd, Bettlach, Switzerland) [15, 17]. No pulse lavage was used.

Key aspects of pre- and postoperative care were protocolled to ensure similar perioperative regimens. All patients received cefazolin prophylaxis during 24-h perioperatively and thromboprophylaxis with low molecular weight heparin for 6 weeks postoperative. All patients followed the same rehabilitation regimen, starting on the first day after surgery, with diclofenac, a non-steroidal anti-inflammatory drug (NSAID), except when use was medically contraindicated. For the NSAID use, patients received a 14-day prescription for home use of 50 mg 3 times a day. We did not follow-up the therapy compliance of the patients. No radiotherapy was used to prevent HO.

### Radiological assessment

**Fig. 1:**
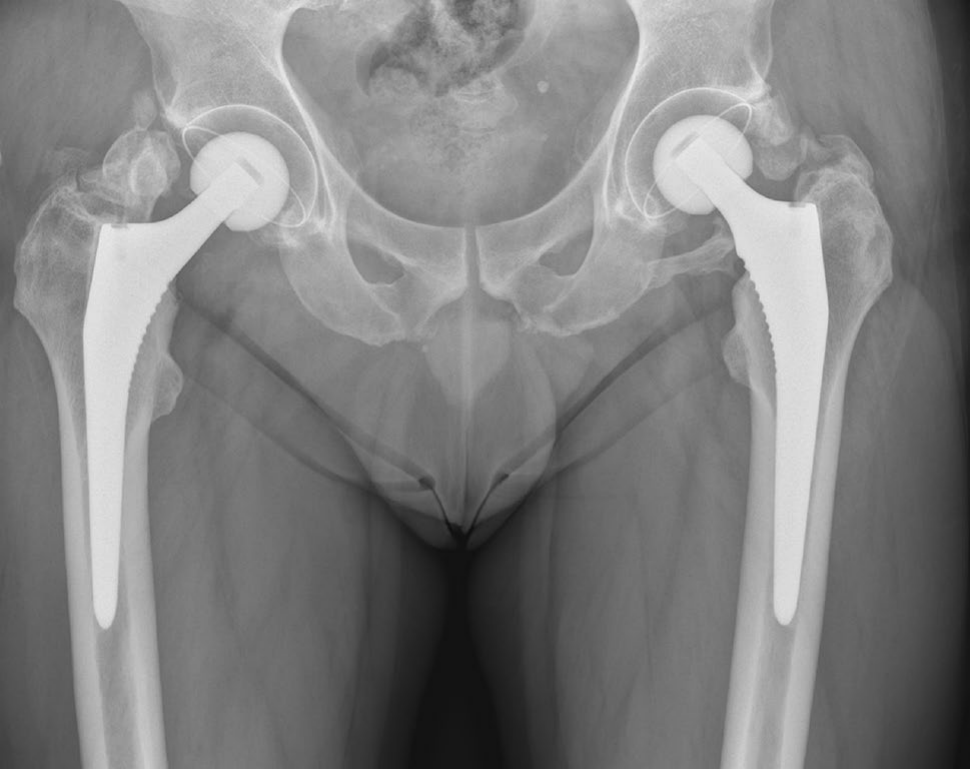
A patient with severe, symptomatic, bilateral HO after THA

The Brooker classification was used for grading of HO, which has proven to be the most accurate scoring system for HO [18]. Figure 1 shows an example of a patient with Brooker grade 4 HO. Radiological assessment was performed twice by two authors independently (JRAM and ST). The authors were blinded for the approach. Brooker grade 1–2 was considered as radiological mild HO and Brooker grade 3–4 was considered as radiological severe HO.

### Statistical analysis

Statistical analysis was performed using SPSS Statistics, version 23.0 (IBM Corp., Armonk, NY, USA). Normal distribution of the data was analyzed using the Kolmogorov–Smirnov test. Patients’ characteristics were compared using an independent *t* test and a Chi-square test. The prevalence of HO was compared using a Chi-square test. Clinical outcomes were compared using an independent *t* test.

### Ethical standards

The procedures performed in this study, involving human participants were in accordance with the ethical standards of the institutional and/or national research committee and with the 1964 Helsinki Declaration and its later amendments or comparable ethical standards.

## Results

**Fig. 2:**
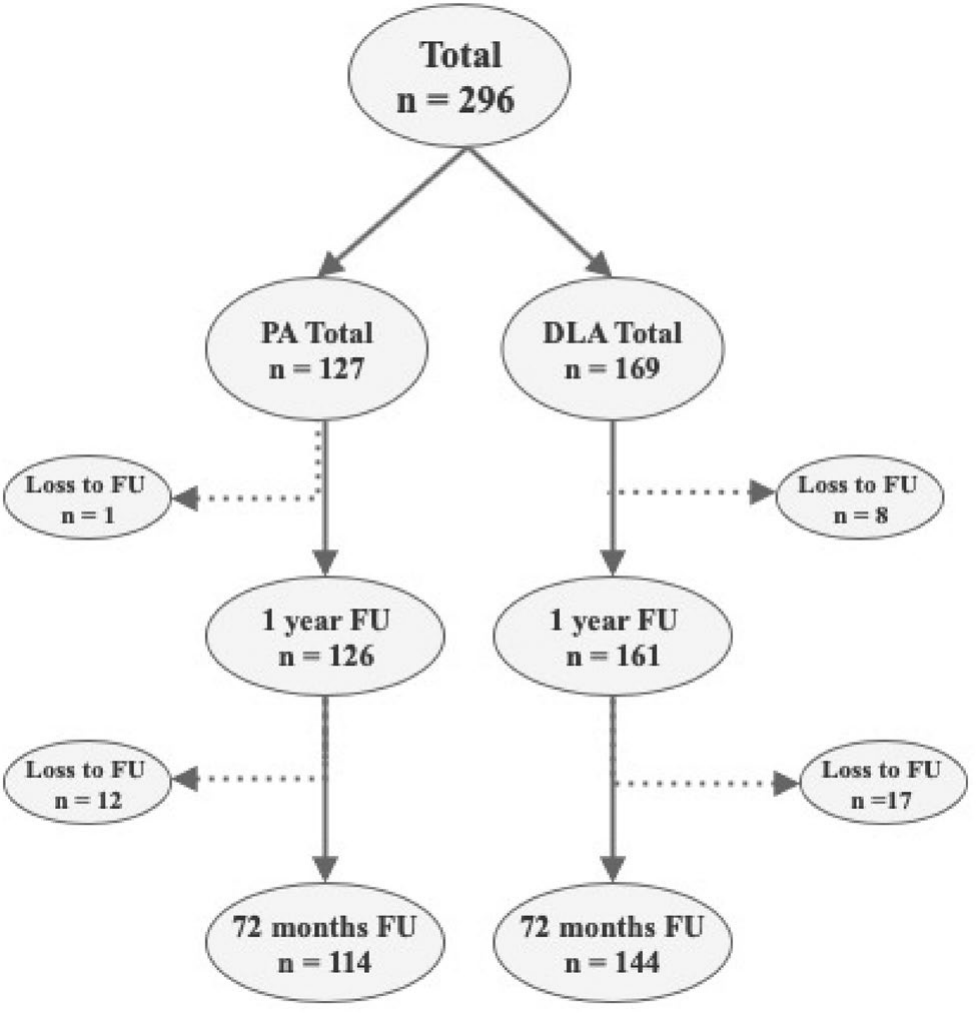
Follow-up flow chart

A total of 258 patients (87%) completed the 6-year follow-up (Fig. 2). Data were normally distributed. A total of 38 patients (13%) were lost to follow-up because of revision, other diseases, unable to attend or death. There were no revisions performed because of HO.

### Heterotopic ossifications

After 1 year, HO was found in 18 (14%) patients of the PA group and in 30 (19%) patients of the DLA group (*p* = 0.207) (Table 2). After 6 years, HO was found in 21 (18%) patients of the PA group and 43 (30%) patients of the DLA group (*p* = 0.024).

**Table 2:** The presence of heterotopic ossifications

	PA (*n* = 126)	DLA (*n* = 161)	*p* value
1 year (%)			
Brooker ≥ 1	18 (14)	30 (19)	0.207
Brooker 3–4	3 (2)	3 (2)	0.537

	PA (*n* = 114)	DLA (*n* = 144)	*p* value
6 year (%)			
Brooker ≥ 1	21 (18)	43 (30)	0.024
Brooker 3–4	5 (4)	7 (5)	0.551

When considering Brooker (3–4) as radiological severe, in both groups three patients (2%) had HO after 1 year (*p* = 0.537) (Table 2). After 6 years, this was 5 (4%) patients in the PA group and 7 (5%) in the DLA group (*p* = 0.551).

### Clinical outcomes

**Fig. 3:**
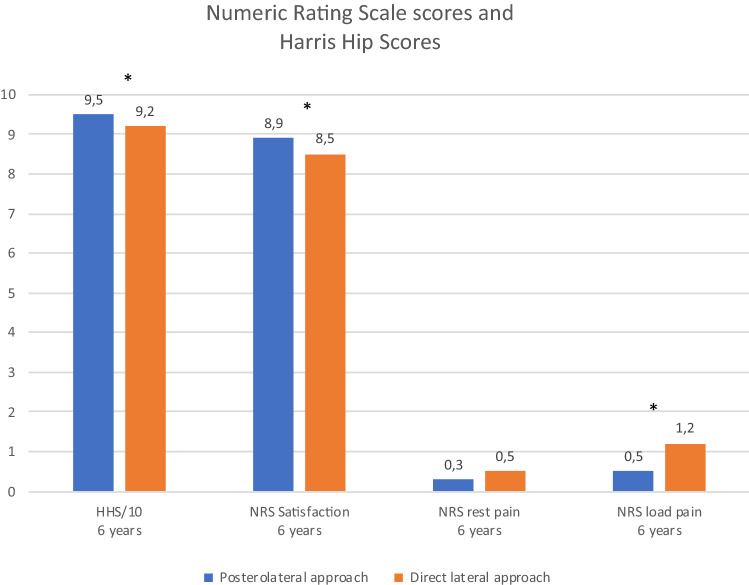
Numeric Rating Scale scores and Harris Hip Scores after 6 years. *Statistically significant difference (*p* ≤ 0.01)

NRS score and Harris Hip Scores (HHS) at 1- and 6-year follow-up are presented in Fig. 3. Scores for both groups at 6-year follow-up are specified in Table 3. After 6 years, the HHS was statistically significant higher after the PA (95.2) compared to the DLA (91.6) (*p* < 0.001). Furthermore, NRS score for patient satisfaction was significantly higher in the PA group (8.9) compared to the DLA group (8.5) (*p* = 0.003). The NRS score for rest pain was equal: 0.3 in the PA group and 0.5 in the DLA group. The NRS score for load pain was statistically significant lower in the PA group (0.5) compared to the DLA group (1.2) (*p* = 0.004) (Table 3).

**Table 3:** Harris Hip Scores (HHS) and Numeric Rating Scale (NRS) scores for patient satisfaction and rest and load pain after 6-year-follow-up

	PA (*n* = 121)	DLA (*n* = 149)	*p* value
Harris Hip Score	95.2 ± 7	91.6 ± 11	< 0.001
NRS for satisfaction	8.9 ± 1	8.5 ± 2	0.003
NRS for rest pain	0.3 ± 1	0.5 ± 1	0.160
NRS for load pain	0.5 ± 1	1.2 ± 2	0.004

After 6 years, in patients with severe HO (Brooker 3–4), the mean HHS was 96.4(± 6) for the PA and 88.5(± 6) for the DLA. The mean NRS for patient satisfaction was 8.8(± 1) for the PA and 8.5(± 1) for the DLA. The NRS for rest pain was 0.0 in both groups. The NRS for load pain was 0.0(± 0) in the PA and 1.5(± 2) for the DLA. For one patient, who underwent the DLA, clinical data were missing (Table 4).

**Table 4:** Mean NRS and Harris Hip Scores in patients with severe HO (Brooker 3–4) after 6 years

	Total mean (SD)	PA mean (SD) *n* = 5	DLA mean (SD) *n* = 6
Harris Hip Score	92.5 (7)	96.4 (6)	88.5 (6)
NRS for satisfaction	8.6 (1)	8.8 (1)	8.5 (1)
NRS for rest pain	0.0 (0)	0.0 (0)	0.0 (0)
NRS for load pain	0.8 (2)	0.0 (0)	1.5 (2)

## Discussion

This prospective cohort study was conducted to compare HO formations between patients who underwent THA either with a PA or DLA. After 6 years of follow-up, statistically significant more radiological HO formation was found after the DLA compared to the PA (30% vs. 18%). However, no difference in radiological severe HO (Brooker 3–4) was found between groups at 6-year follow-up.

The finding that patients undergoing DLA for THA form more HO can be explained by the fact that DLA presumably causes more tissue damage (because this approach requires dissection of gluteal musculature from the trochanter major with specific placement of retractors necessary to perform DLA) [19]. More tissue damage causes more inflammation, and subsequently more HO formation [20, 21].

Some studies have been conducted about the influence of approach of THA on HO formation [6, 12, 14]. However, only one discussed HO formation after PA. This study did not find differences in HO formation (Brooker 1–4) between the anterolateral approach and the PA [13]. However, similar to our study, the lowest incidence of both HO formation (Brooker 1–4) and severe HO formation (Brooker 3–4) was found after PA. Furthermore, nowadays, the direct anterior approach (DAA) is performed in increasing numbers. Alijanipour et al. compared DAA with DLA and found a statistically significant more HO formation in DLA [6]. However, no statistically significant difference was found in the severe HO formation between DAA and DLA. Zran et al. found a lower incidence of HO in patients undergoing a PA (27.6%) compared to patients undergoing DLA without orthopedic table (47.7%) (*p* < 0.01) [22]. They, as well, did not find significant differences regarding the severity of HO.

At the 6-year follow-up, we found significantly better HHS and NRS scores for patient satisfaction and load pain for patients who underwent PA compared to DLA. In our opinion, these results cannot be attributed to less HO formation in the PA group compared to DLA because more factors related to the surgical approach may influence outcome of THA [23]. Overall, clinical scores for patients with severe HO were evidently lower compared to the total patient group. However, differences between the approaches could not be tested due to the small patient number.

It is clear that severe HO formation decreases clinical outcomes. However, further research with a higher number of patients with severe HO formation is necessary to define the effect of surgical approach on severe HO formation since a low Brooker grade (1–3) does not seem to impact outcomes [24]. However, given the fact that no revisions for HO were performed in our cohort, the clinical relevance of HO formation can be questioned. Nevertheless, this knowledge can be of interest for clinical decision making and future studies in the field of THA approaches since it seems that more tissue damage is associated with more HO formation. Especially in patients with pre-existent risk factors for the formation of HO, one could consider minimizing the risk of developing HO when choosing the surgical approach.

This is the first large, long-term, prospective cohort study that compares the prevalence of HO between these two approaches. However, this study has some limitations. First, only two approaches were compared. Second, no randomization was performed. However, all measurements were performed twice by two authors who were blinded for surgical approach. Nevertheless, it was not possible to correct for unknown confounding factors that may affect HO formation. Third, the data on duration of NSAID use were not optimal. Long-term NSAID use may influence the (amount of) HO formation [25, 26]. However, there is no reason to assume the use of NSAIDs was different between groups. Furthermore, Haffer et al. showed that diclofenac is efficacious in the prevention of HO when started the first day postoperative for a minimum of 9 days [27]. In the Netherlands, all hospitals follow standard protocols for postoperative NSAID use with at least a 14-day prescription.

The current study shows the difference in HO formation after THA with PA or DLA. The good clinical scores and high follow-up rate show that the THA technique used in the current study is functioning well. Due to the presence of several confounding factors associated with the different approaches, this study cannot draw any conclusions with regard to the influence of HO formation on clinical outcomes. Due to the small number of patients with severe HO formation, this study cannot draw any conclusions with regard to the influence of approach on severe HO formation. A large, long-term randomized controlled trial comparing approaches can assess with which approach most (severe) HO formation occurs. The resulting frequencies of HO formation and clinical outcome scores can eventually determine the influence of the different approaches in THA.

In conclusion, this study shows that DLA for THA is associated with more radiological HO formation compared to the PA. However, there is no difference in the prevalence of radiological severe HO. No revisions were performed because of HO within the 6-year follow-up after THA. Long-term clinical trials are needed to assess the influence of approach on HO formation and clinical outcome scores.
